# Effect of wetting agent coverage on the surface properties of resin composite submitted to brushing and staining cycles

**DOI:** 10.4317/jced.58311

**Published:** 2021-08-01

**Authors:** Pâmela-Letícia Pereira, Renata Pereira, Bruna-Guerra Silva, Rodrigo-Barros-Esteves Lins, Débora-Alves-Nunes-Leite Lima, Flávio-Henrique-Baggio Aguiar

**Affiliations:** 1 Undergraduate student, Department of Restorative Dentistry, Division of Operative Dentistry, Piracicaba Dental School, University of Campinas (UNICAMP), Av. Limeira, 901, Zip code 13.414-903, Piracicaba, SP, Brazil; 2 PhD student, Department of Restorative Dentistry, Division of Operative Dentistry, Piracicaba Dental School, University of Campinas (UNICAMP), Av. Limeira 901, Zip code 13.414-903, Piracicaba, SP, Brazil; 3 Substitute Professor, Department of Restorative Dentistry, Paraiba State University (UEPB), Rua Horácio Trajano de Oliveira 666, Zip code 58070-450, João Pessoa, PB, Brazil; 4 Associate Professor. Department of Restorative Dentistry, Division of Operative Dentistry, Piracicaba Dental School, University of Campinas (UNICAMP), Av. Limeira, 901, Zip code 13.414-903, Piracicaba, SP, Brazil

## Abstract

**Background:**

Wetting agents facilitate the composites handling, acting as a lubricant and decreasing their stickness to spatula. The effects of these materials on the properties of composites are not completely clear. This study aimed to evaluate Gloss, Color, Roughness and Microhardness of a composite (Filtek Z250 XT, 3M Oral Care) covered by a wetting agent (Modeling Resin, Bisco), submitted to brushing and staining cycles with red wine.

**Material and Methods:**

Cylinder-shaped samples (8 mm ø x 2 mm height) were divided into 4 groups, according to application of wetting agent and brushing cycles (n = 20). The composite was placed in the orifice of a polytetrafluoroethylene mold, received wetting agent coverage, and was light-cured. Gloss, Color, Roughness and Microhardness were evaluated in two times: after samples confection and after brushing + staining cycles. Data were submitted to one-way ANOVA and Tukey’s test (ΔL, Δa, Δb and ΔE) and two-way repeated measures ANOVA and Bonferronis’s test (gloss, roughness and microhardness).

**Results:**

In general, the composite showed higher gloss values when added by the wetting agent. No statistical differences were observed regarding colors’ groups. Roughness increased after brushing cycles, regardless of wetting agent application. The only group which presented decreased Microhardness after cycles was the group without wetting agent, only submitted to staining with red wine.

**Conclusions:**

The application of wetting agent on the composite did not interfere negatively with its properties of Gloss, Color, Roughness and Microhardness.

** Key words:**Resin composite, wetting agent, surface properties, gloss, color, esthetic dentistry.

## Introduction

From self-cure polymethyl methacrylate (PMMA) to bulk-fill resins, dental composites have faced great evolution through the last decades (1). In an era in which information is easily reached, society becomes more demanding in relation to restorative treatments (2,3). At the same time, besides requiring development of the composites properties, dental professionals claim for easily handling materials to save chairside time and increase productivity. As result, dental industry has been investing in the creation of new materials, which meet the demands of the population and the convenience of professionals (4).

In this scenario, the wetting agents for resin composites were released on the dental market by Ultradent, GC, Kerr, Bisco and others. Based on flowable methacrylate-based composites and presenting range of 30 to 45% of filler weight, manufacturers argue that such materials facilitate the composites handling, acting as a lubricant and decreasing their stickness to spatula, helping their adaptation to the cavities and favoring the process of sculpture and definition of restorations margins.

The launch of such agents do not date older than 2005, howbeit the concept of lubricating the instrument to favor the handling of resin composites is more longevous. Since late 1980’s, reports relate the use of acetone, isopropyl alcohol or adhesive systems within layers of resin composite with keeping or increasing of its interlayer or cohesive bond strength and flexural strength (5-7). Surface properties were also tested and the use of adhesive systems as lubricants suggested decrease of color change and staining process over time (8,9).

Barcellos *et al*. (10) were the first who tested also a wetting agent, specifically designated to act as a modeling resin for direct composite restoration, other than just solvent or adhesive system. Similarly to the previous studies, they found out that lubricating instruments with such agents do not reduce the cohesive strength of resin composite. Supplementarily, in this case the advantage of the so-called wetting agents is that, unlike some adhesive systems, they are free of 2-hydroxyethyl methacrylate (HEMA), minimizing water sorption, incomplete polymerization and osmotic breakdown (11).

The proposal of the wetting agents is worthy of attention and, apparently, it facilitates the restorative procedure with resin composite. However, the implications of these materials for the properties of composites are not completely clear. Hitherto, the studies of such agents are scarce (10,12,13). None of them tested composites’ gloss, one of the properties deemed most positively affected by the wetting agent use. Moreover, to the best of our knowledge, brushing effects were not previously considered. Thus, further studies are necessary to analyze the effects of wetting agents on the properties of resin composites, in order to indicate their safe use during clinical practice. The aim of this *in vitro* study was to evaluate Gloss, Color, Roughness and Microhardness of a mycrohibrid conventional resin composite (Filtek Z250 XT, 3M Oral Care, St. Paul, MN, USA), commonly used in clinical practice (14), covered or not by a wetting agent (Modeling Resin, Bisco, Schaumburg, IL, USA), submitted to brushing and/or staining cycles with red wine.

## Material and Methods

-Samples confection

Samples were divided into 4 groups (n=20), according to application of wetting agent and brushing cycles: G1-Resin composite; G2-Resin composite submitted to brushing cycles; G3-Resin composite covered by wetting agent; G4-Resin composite covered by wetting agent submitted to brushing cycles. Considering Microhardness evaluation alter permanently the sample surface, 10 samples of each group were evaluated regarding Gloss, Color and Roughness and further 10 were evaluated regarding Microhardness.

A description of materials used is presented on Table 1. For samples confection, a 2mm increment of resin composite (Filtek Z250 XT, A2 Shade) was placed into the orifice of an individualized polytetrafluoroethylene cylinder-based mold (8 mm ø x 2 mm height) with the aid of a spatula for composite filling (Suprafill 1, Duflex, SS White, Rio de Janeiro, RJ, Brazil). Right after the composite placement, the wetting agent (Modeling Resin, Bisco) was dispensed in a glass dappen dish, the spatula immersed the material for 3 seconds and applied over the samples of groups 3 and 4, adapting the resin composite to the mold. A polyester strip, followed by a glass cover slip, were then placed over the mold and pressed with a 500 g load for 1 minute to ensure compaction and prevent void development within the uncured composite. The glass cover slip was removed and samples were light-cured for 20 seconds (Valo, Ultradent Products Inc., S. Jordan, UT, USA) in Standard mode: 1000 mW / cm² (20 J/cm2). The tip was positioned directly over the polyester strip and the curing power was previously verified (Ophir Optronics, Jerusalem, Israel). Samples were incubated for 24 hours at 37°C and 100% relative humidity. Afterwards, they were polished at low-speed with the complete system of 13 mm aluminium oxide-based Sof-Lex polishing discs (3M Oral Care), for 30 seconds each disc. The samples were rinsed after each polishment for 10 seconds. After 24 hours, the following initial evaluations were performed.

**Table 1:**
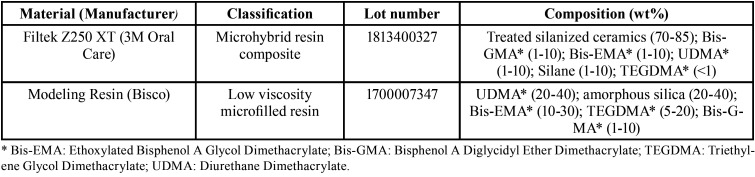
Materials specifications as reported by manufacturer.

-Gloss evaluation

A glossmeter (ZGM 1120, Zehntner Testing Instruments, Sissach, Switzerland) was used to measure the gloss values of the samples surfaces at 60° of light beam incidence, according to ISO 2813:2014 (15). A metallic holder device kept the samples protected from external light intervention. Each sample was moved three consecutive times at 120º to make three measurements. The values’ mean was recorded as the mean Gloss Unit (GU) of each sample surface.

-Color evaluation

Each sample was hold by a polytetrafluoroethylene-based device, which was taken to a light chamber (GTI MiniMatcher MM-1, GTI Tecchnology, Newburgh, NY, USA), previously set to ambient light condition. Samples were then evaluated through spectrophotometer (CM 700D, Minolta, Osaka, Japan), according to the manufacturer’s instructions. The color values were quantified by On Color QC Lite software (Konica Minolta, Chiyoda, Tokyo, Japan) and presented through CIE L* a* b* system, in which L* indicates variations of lightness (black-white), while a* and b* indicate variations of chromes (red-green and yellow-blue, respectively). The values’ mean of three measurements was calculated for each sample.

-Roughness evaluation

Surface roughness was measured by a perfilometer (Surftest 211, Mitutoyo Corp., Tokyo, Japan). Each sample was positioned parallel to the equipment surface and perpendicularly to the profile tip. Roughness values were obtained at 0.05 mm/s using cut-off of 0.25mm at three equidistants points on the sample surface. The mean of the three measurements was recorded as the mean Roughness (Ra) of each sample surface.

-Knoop Microhardness evaluation

Microhardness was evaluated on the top surface of each sample, using a Microhardness tester (HMV-2000, Shimadzu Corporation, Tokyo, Japan) with a Knoop diamond indenter. Three equidistant measurements were performed under 50g load for 15 seconds on the surface of each sample. The values’ mean was recorded as the mean Knoop Microhardness (KHN) of each sample surface.

-Brushing cycles

After initial evaluations, samples of groups 2 and 4 were submitted to brushing cycles in a simulated tooth brushing machine (MSEt, Biopdi, São Carlos, SP, Brazil), following ISO/TS 14569-2 and ISO 11609:2010 specifications (16,17). One soft toothbrush (Oral B Indicator Plus, Procter & Gamble, Cincinnati, OH, USA) was used for each sample. Initially, toothbrush head was sectioned from the handle part with a double-sided diamond disc (KG Sorensen Ind. Com. Ltda, Barueri, SP, Brazil). Each head was then fixed to the machine’s toothbrush holder through thermoplastic glue (Brascola, São Bernardo do Campo, SP, Brazil), so that the toothbrush bristles contacted directly the sample surface.

The equipment provided linear brushing movements through the samples surfaces at 120 cycles per minute and 37°C. The surface of each sample was submitted to 10000 brushing cycles at frequency of 2.5 Hz under 200 g vertical load with an abrasive mixture, simulating one year of clinical situation (18). The abrasive mixture consisted of fluoride dentifrice (Colgate Triple Action, Colgate-Palmolive Ind. E Com. Ltda., São Paulo, SP, Brazil) and distilled water, in proportion of 1: 3 by weight. At the end of the brushing cycles, the samples were removed from the equipment, rinsed with distilled water and dried with soft tissue paper.

-Staining cycles with red wine

All samples were immersed in red wine (Cigarra TTO, Casa Santos Lima, Lisbon, Portugal) for 5 minutes during 7 days. After each immersion, they were rinsed for 30 seconds in running water to remove any possible wine sediments. Between the immersion periods, the samples were stored in distilled water at 37ºC. At the end of staining cycles, the samples were washed for 5 minutes and dried with soft tissue paper.

-Final evaluations and Statistical analyses

After brushing and/or staining cycles, according to each group, samples were submitted to final evaluations of surface gloss, color, roughness and microhardness following the methodology previously mentioned. For color evaluation, the difference between initial and final L*, a*, and b* values was obtained (ΔL, Δa, and Δb). Total color change (ΔE) was calculated according to the formula: ΔE = [(L1 - L0)2+ (a1 - a0)2 + (b1 –b0)2] ½, where L1, a1 and b1 were considered the final values of lightness and red-green and yellow-blue chromes. Statistical analyses were performed using SPSS 21.0 Software (IBM SPSS Statistic for Windows, v. 21.0, IBM Corp., Armonk, NY, USA), with a significance level set at 5%. Normality and homoscedasticity of data were confirmed through Shapiro-Wilk and Levene tests (*p* > 0.05). One-way ANOVA and Tukey’s post-hoc test was performed for color parameters (L*, a*, b* and ΔE). Gloss, surface roughness and microhardness were analyzed by two-way repeated measures ANOVA and Bonferroni’s post-hoc test.

## Results

**Figure 1:**
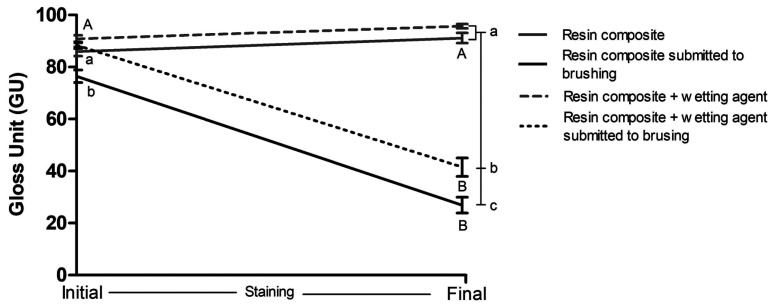
Gloss (GU) of resin composite, covered or not by wetting agent, over time. Caption: Means and standard deviations followed by distinct letters differ from each other (p≤0.05). Uppercase letters compare gloss before and after brushing and/or staining cycles, while lowercase letters compare gloss between groups.

Figure 1 presents the graph of gloss results. Groups that underwent only staining cycles presented statistically similar gloss values at initial and final times (*p* > 0.05), regardless of having coverage with wetting agent. Groups submitted to brushing and staining cycles had their gloss decreased (*p* < 0.001), regardless of having coverage of wetting agent. At initial time, resin composite covered by wetting agent presented higher gloss than the not covered one (*p* < 0.001). At final time, the groups only submitted to staining, regardless of having coverage of wetting agent, showed the highest gloss values (*p* < 0.001), followed by the group covered by wetting agent submitted to brushing and staining cycles. The group not covered by wetting agent submitted to brushing and staining cycles was the one that showed the lowest gloss values (*p* < 0.001).

No statistical differences were observed regarding color parameters change (ΔL, Δa, and Δb) and total color change (ΔE) of the groups investigated (*p* > 0.05) (Table 2).

**Table 2:**
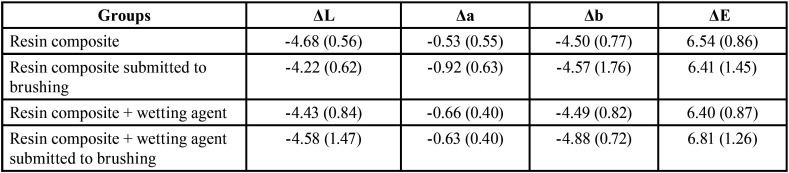
Mean (standard deviation) Color change (ΔL, Δa, Δb, ΔE) of resin composite covered or not by wetting agent after brushing and/or staining cycles.

**Figure 2:**
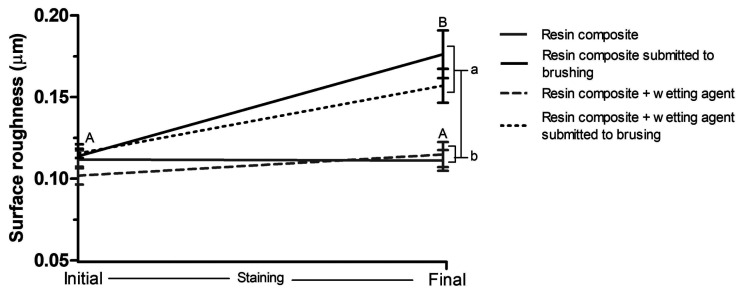
Roughness (Ra) of resin composite, covered or not by wetting agent, over time. Caption: Means and standard deviations followed by distinct letters differ from each other (p≤0.05). Uppercase letters compare roughness before and after brushing and/or staining cycles, while lowercase letters compare roughness between groups.

Figure 2 shows the graph of roughness results. Groups that were submitted to brushing and staining cycles, regardless of being covered by wetting agent, had their roughness increased after the cycles (*p* < 0.001), while the values obtained by the groups that went only through staining remained statistically similar (*p* > 0.05). At initial time, all groups showed statistically similar roughness values (*p* > 0.05). At final time, the groups that went through brushing and staining cycles showed higher roughness values when compared to those that were only submitted to staining (*p* < 0.001).

**Figure 3:**
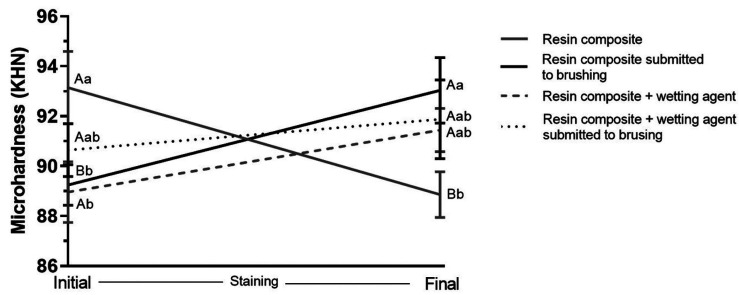
Microhardness (KHN) of resin composite, covered or not by wetting agent, over time. Caption: Means and standard deviations followed by distinct letters differ from each other (p≤0.05). Uppercase letters compare microhardness before and after brushing and/or staining cycles, while lowercase letters compare microhardness between groups.

Figure 3 presents the graph of microhardness results. The only group that had its microhardness decreased after the cycles was the group not covered by wetting agent (*p* = 0.013), which underwent only staining. On the other hand, the only group that had its microhardness increased was also the group not covered by wetting agent, but also submitted to brushing (*p* = 0.026). The groups covered by wetting agent presented statistically similar microhardness results at initial and final times (*p*> 0.05). At initial time, the group not covered by wetting agent obtained higher initial microhardness when compared to the group covered by wetting agent (*p* < 0.022). At final time, the group not covered by wetting agent, submitted to brushing and staining cycles, presented higher microhardness values than the group not covered by wetting agent that went only through staining cycles (*p* < 0.015). The groups covered by wetting agent did not differ statistically from the others (*p* = 0.05).

## Discussion

The wetting agents for resin composites were launched onto the dental market in order to favor the handling of composites, eliminating their adherence to spatula, and facilitating placement, as well as restoration sculpture. Although adhesive systems or pure solvents have been previously indicated for the same purpose in the so-called Restorative Dental Modeling Insertion Technique (RDMIT), except for Barcellos *et al*. (10), who tested Composite Wetting Resin (Ultradent Products Inc.), Tuncer and colleagues (12), who tested Modeling Resin (Bisco) and Kutuk *et al*. (13), who tested Modeling Liquid (GC Corp., Tokyo, Japan), no further researchers investigated the use of wetting agents and its implications on resin composites properties. Considering that such agents are applied directly on the last layer of resin composite during restoration procedure and therefore the composites’ surface properties could be altered, it is evident that the study of their coverage effects on resin composite is essential. Thus, this study aimed to analyse the effects of a wetting agent (Modeling Resin, Bisco) on gloss, color, roughness and microhardness of a conventional resin composite, submitted to brushing and/or staining cycles.

All the samples submitted to brushing and staining had their gloss decreased and roughness increased after the cycles, regardless of being covered by wetting agent. The results indicate that the wetting agent was not capable to totally suppress the deleterious effects of brushing. Nevertheless, the findings related to the brushing process itself are in compliance with previous studies, which indicate inverse correlation between roughness and gloss (19). The dentifrice abrasive content associated with brushing process possibly worn the composite organic matrix more quickly than the load particles, resulting in rougher and less glossy surface. The samples submitted only to the staining process maintained statistically similar gloss and roughness after the procedure. As explained by Ruivo *et al*. (20), as well as by Ferretti and collegues (14), when composites are submitted to brushing, chewing and other stresses, the organic matrix is abraded, leading to particles protrusion or removal, which increases the peak-valley distance and leads to development of cracks, resulting in greater surface roughness. The coverage by wetting agent, in this case, did not make an exception to the rule, especially because of the high organic matrix and lower particle content of the agent. Namely, the agent’s organic matrix was normally abraded during the brushing procedure. One may infere that in a daily situation, with the composite being submitted to constant stresses, the agent is gradually removed.

Interestingly, the alcohol content associated with the low pH (3.46) of red wine alone was not enough to promote possible chemical degradation of organic matrix, affect the surface integrity of the composite and alter its roughness and gloss, as it could be inferred considering previous studies findings (21,22). One may suggest that duration of staining cycles was not long enough to cause such negative effects, since any possible impacts of wine on resin composite is time-dependent (23). The same explanation may be applied to justify the color results. No statistical differences were observed regarding color change of the groups investigated. Such finding corroborates those from previous researches, in which staining with red wine had no significant influence on the color stability of restorative materials (24), or 1 week of wine storage did not affect the color variation of all composites (25).

It is worthy of attention the gloss values obtained by the groups covered by wetting agent in comparison to the not covered ones. Albeit brushing and staining cycles led to gloss decrease, at final time the goup covered by wetting agent showed higher gloss values than the group without wetting agent coverage, both submitted to the same procedures. Conversely, the group of resin composite without wetting agent submitted to brushing and staining cycles was the one that showed the lowest gloss values. Gloss is defined as the specular reflection obtained from a surface compared to a standard one, i.e. black glass (26). Its values may be resulted from interactions of several factors, such as the material composition, its degree of conversion, type and size of load particles, as well as differences of refractive indexes of organic matrix and particles (19,27). Modeling Resin is a low viscosity microfilled resin, composed by 30% by weight of amorphous silica. Considering light attenuation through composites is result of a process of scattering and absorption by constituents, such as monomer, pigments, and fillers (28), the results obtained by the groups covered by wetting agent may lie in the lower particles content of the agent. It turns out that the fewer particles, the lower the interference for light attenuation, the more homogeneous its reflection, so the higher the gloss. Additionally, the application of the wetting agent itself, as advocated by the manufacturer, favors the composite handling, possibly decreasing its viscosity. Peutzfeldt and Asmussen (29) showed that degree of fluidity when applying the composite influences gap formation. A more homogeneous and glossy surface should therefore be expected.

Although no reports are found in literature about the impact of application of weting agent, specifically, on resin composites gloss, the study of Sedrez-Porto and colleagues (9) may reinforce our findings. The authors showed that, when stored in wine, the composite surface degradation was less intense for specimens prepared with adhesive systems than for specimens without it. The result was correlated to the hydrophobic composition of the adhesive systems, which possibly protected the composite from hydrolysis and further deleterious effects.

The important role of hydrophobicity of bonding agent used as instrument lubricant to protect resin composites is no longer a new finding (30). The same explanation, added to the fact that Modeling Resin is HEMA-free, may also justify our results of microhardness. From the groups only submitted to staining cycles, the one which received wetting agent application did not show decreased microhardness at final time, whilst the the group not covered by wetting agent presented opposite result. Phase separation may occur within HEMA-free resin systems. The gradual evaporation of the solvent leads to phase-separation reaction, in which water separates from other ingredients. Such reaction may be regarded as beneficial, as they prevent water to get trapped in the composite layer and cause osmotic breakdown (11). In case of the group not covered by wetting agent, besides not being protected by a hodrophobic-like material, the alcohol content associated with the low pH of red wine might have contributed, albeit at very subtle levels, to plasticize the polymer matrix and soft the composite surface (21). When it was also submitted to brushing, though, as already elucidated, the organic matrix was abraded and got further softer, leading also to particles protrusion or removal (14,20). The exposed hard particles of zirconia presented by Filtek Z250 XT led then to increase of microhardness. Likewise, previous studies reported similar results (14,19). Such finding point out again the impact of the composite protection by the wetting agent, so much so that the microhardness was maintained for the group covered by wetting agent and submitted to brushing cycles.

## Conclusions

When covered by wetting agent, the resin composite gloss and microhardness were more preserved from consequences of immersion in red wine and wear-related brushing. The findings, however, suggest that in daily situation, such positive effects are time-limited, once the wetting agent was not capable to totally suppress the deleterious effects of brushing. Yet, this study supports evidence that wetting agent does not jeopardize the surface properties of a conventional resin composite, and its influences, when present, are short-term positive.
